# The Association Between Regular Physical Exercise, Sleep Patterns, Fasting, and Autophagy for Healthy Longevity and Well-Being: A Narrative Review

**DOI:** 10.3389/fpsyg.2021.803421

**Published:** 2021-12-02

**Authors:** Sicheng Min, Bojan Masanovic, Te Bu, Radenko M. Matic, Ivan Vasiljevic, Marina Vukotic, Jiaomu Li, Jovan Vukovic, Tao Fu, Blazo Jabucanin, Rajko Bujkovic, Stevo Popovic

**Affiliations:** ^1^College of Physical Education, Hunan Normal University, Changsha, China; ^2^Faculty for Sport and Physical Education, University of Montenegro, Niksic, Montenegro; ^3^Montenegrosport, Podgorica, Montenegro; ^4^Faculty of Sport and Physical Education, University of Novi Sad, Novi Sad, Serbia; ^5^Western Balkan Sport Innovation Lab, Podgorica, Montenegro; ^6^College of Exercise and Health Sciences, Tianjin University of Sport, Tianjin, China; ^7^Faculty of Business Studies, Mediterranean University, Podgorica, Montenegro; ^8^Montenegrin Sports Academy, Podgorica, Montenegro

**Keywords:** exercise, sleep, fasting, autophagy, health, longevity, well-being

## Abstract

This narrative review of the literature assessed whether regular physical exercise and sleep patterns, fasting and autophagy, altogether can be an adequate strategy for achieving healthy longevity and well-being within different stage of life. There are a large number of studies dealing with well-being and healthy longevity; however, few of them have given us a specific formula for how to live long and healthy. Despite all the advances that have been made to create adequate physical exercise programs, sleep patterns or nutritional protocols, the relation between different types of fasting, nutritional supplementation as well as regular physical exercise and sleep patterns have not yet been satisfactorily resolved to cause the best effects of autophagy and, therefore, well-being and healthy longevity. In this way, future studies should clarify more efficiently the relationship between these variables to understand the association between regular physical exercise, sleep patterns, fasting and autophagy for healthy longevity and well-being.

## Introduction

The twentieth century is characterized as the century of world population growth and it is considered one of the most significant achievements of civilization, while the twenty-first century is characterized as the century of aging. The global average life expectancy was extended by 5.5 years in the period from 2000 to 2016 (Vukovic, 2021), which is the largest increase since the 60s of the twentieth century. According to United Nations projections, the number of people over the age of 60 will more than double by 2050, from the current 840 million to over 2 billion people (Vukovic, 2021).

There are a large number of studies dealing with healthy longevity and well-being; however, few of them have given us a specific formula for how to live long and healthy. Yet there is one man who has brought us closer to our goal, and that is Yoshinori Ohsumi, a 2016 Nobel Prize-winning researcher in Physiology or Medicine for his initial interpretation of the mechanisms of autophagy (Antunes et al., 2018). Although the contributions of this great scientist are very well-known in the field of science, the global public, unfortunately, is not adequately acquainted with his conclusions and recommendations, as well as the outputs of a large number of studies that followed his work. For this reason, this narrative study aimed to point out that an adequate physical activity, sleep patterns, fasting, and autophagy may be highly correlated with to healthy longevity and well-being.

According to Antunes et al. (2018), autophagy is “an evolutionarily conserved lysosomal catabolic process by which cells degrade and recycle intracellular endogenous (damaged organelles, misfolded or mutant proteins and macromolecules) and exogenous (viruses and bacteria) components to maintain cellular homeostasis.” Therefore, autophagy has, lately, been used in solving various health problems, including one of the greatest enemies of longevity such as cancer (Galluzzi et al., 2015; Amaravadi et al., 2016), and one of the global epidemic and the most blatantly visible yet neglected public health problem nowadays such as obesity (Lavallard et al., 2012), as well as infections and inflammatory diseases (Cadwell, 2016), neurodegeneration (Menzies et al., 2017), metabolic and cardiovascular diseases (Bravo-San Pedro et al., 2017), et cetera. Thus, it is not wrong to assume that autophagy represents well-trodden path in lifespan extension and well-being, as well as it is the primary mechanism that can help removing damaged organelles, such as mitochondria, which may have a direct impact on aging (Yen and Klionsky, 2008). On the other hand, it is very important to emphasize that regular physical activity, sleep patterns and proper nutrition are also important factors in achieving healthy longevity and well-being. So, the main goal of this study is to offer basic knowledge of association between regular physical exercise, sleep patterns, fasting and autophagy in the process of achieving healthy longevity and well-being through narrative review of scientific literature.

## Physical Exercise

It is well-known that physical exercise, defined as “planned, structured and repetitive physical activity, has been an important force to alter physiological characteristics of human being during the process of evolution” (cited in Andreotti et al., 2020). On the other hand, physical exercise is well-connected to the aging process. This is reflected in by molecular and cellular changes over a longer period of time and it can lead to the deterioration of physiological parameters important to keeping a human body alive and healthy (cited in Andreotti et al., 2020). So, aging is “a physiological process characterized by a progressive decline of biological functions and an increase in destructive processes in cells and organs, while physical activity and exercise positively affects the expression of skeletal muscle markers involved in longevity pathways” (Mancini et al., 2019). Therefore, it is very important to understand the role of physical exercise in the process of achieving healthy longevity within different stage of life from childhood to old-age.

It is generally known that physical exercise is a natural activity during childhood, but it is very important to emphasize that habits are acquired then, but also improve certain physical abilities and characteristics. Special attention needs to be paid from adolescence onwards. Namely, physical exercise can have beneficial effects on the ability to pay attention and cognitive function in adolescents that are the findings of a study conducted by Vanhelst et al. (2016) and de Greeff et al. (2018). Also, during adolescence, certain cases of depression can occur, and physical exercise has been shown to be an effective factor in reducing the symptoms of depression, both in adolescents and young people, it was found in the studies that were conducted by Balchin et al. (2016), Sadeghi et al. (2016), Radovic et al. (2017), and Bailey et al. (2018). Physical exercise is also useful for the working population, but it is the most useful for the elderly population, who are constantly recommended regular physical exercise. However, few of older people follow the recommendations, especially moderate to vigorous physical exercise; even, some believe that physical exercise could be potentially harmful (Franco et al., 2015). According to Hupin et al. (2015), moderate physical activity can reduce mortality risk up to 22%, so it is not surprising that Cotman and Berchtold (2007) have concluded that “among the various interventions that affect aging, physical exercise seems to be the main ally in the prevention of aging-related diseases” and, therefore, the main ally of healthy longevity and well-being.

In summary, regular physical exercise increases skeletal muscle fat oxidation and anti-oxidative potential, stimulates musculoskeletal metabolic adaptations, and results in beneficial improvements in the cardiovascular system (Andreotti et al., 2020). Also, regular physical exercise improves quality of sleep and increases brain-derived neurotrophic factor production, and can decrease neuronal death and improve cognitive performance (Mancini et al., 2019). Regular physical exercise can also cause many other effects that can affect positive aging and health; however, the molecular effects of exercise training on regulation of autophagy and processes involved in longevity promotion are not completely explained by the scientific society, and there is no general agreement on this issue (Andreotti et al., 2020).

## Sleep Patterns

In regular conditions, people spend about one-third of their life either sleeping or attempting to do so. The key significance of sleeping time is enabling enough time for recovery functions of the organism which is crucial for effects in human health, well-being, longevity, and optimal daytime functioning (Sorathia and Ghori, 2016; Bertozzi et al., 2017). This fact confirmed that enough quality sleep is necessary to help each person maintain other activities in their daily routine. Therefore, it's clear that human health and well-being are associated with regular sleep patterns as an essential factor for 24 h of human functioning and help to the maintenance of homeostatic needs (Tufik et al., 2009). On another side, some estimations highlighted that every third person globally has sleep-related problems (Roth, 2007). These problems with sleep disorders can be caused by different factors, such as physical or medical, psychiatric, aging, genetics, medications, lifestyle, and socio-demographic factors (smoking, exercise, education, age, gender, et cetera). Further, sleep disorders can increase the number of cardiovascular (Basnet et al., 2016) and metabolic diseases (Karthikeyan et al., 2019), which are frequently causes of human diseases and mortality.

Researchers put a lot of effort to reveal contextual factors which are associated with managing sleep-related problems, intending to impact on physical, mental, social, and emotional health and well-being of people. Some conclusions revealed the negative effect of physical load at the job and intensive physical exercise on insomnia (Dubinina et al., 2021). Further, the importance of quality of sleep in a psychological context showed that depression is associated with sleep problems (Basnet et al., 2016). Also, conscientiousness, as a personal trait, can be an essential moderator in the protection of individuals of poor sleep (Williams and Moroz, 2009). On the contrary, human dispositional optimism contributes to a lower risk of chronic insomnia (Weitzer et al., 2021), and regular sleep has significant importance to cognitive health and higher level dopaminergic activity (Yang et al., 2014). Further, Eisenberger and Cole (2012) revealed the importance of social relationships in the same context.

Contemporary managing sleep-related problems require a deep approach through an interdisciplinary and holistic approach. That is a reason why many researchers point out that improvement in managing sleep problems contributes to a higher quality of life, well-being, and longevity in a population (Basnet et al., 2016).

## Fasting

Fasting is “characterized by the complete deprivation of food but not water, with intervening periods of normal food intake” (Mattson et al., 2017). It has been known since ancient times, and it has remained in modern religions as a significant activity in religious rites. Although ancient religious knowledge was not based on adequate scientific facts, it is still interesting to mention it, mostly because it turned out to be correct, and the main question is how ancient people came to such valid knowledge. However, the goal of this study is not to deal with knowledge of ancient people and their methods in reaching it, but to determine contemporary knowledge and methods that would improve the health status of contemporary people and prolong their lives. Therefore, in the description of current knowledge of fasting, it is important to highlight that, according to the suggestions of Mattson et al. (2017), it has been found that fasting can “improve cardiometabolic health, reduce cancer incidence, reduce the rate of tumor growth, regenerate organs by increasing stem cell production, and increase lifespan,” which significantly leads to the conclusion that it has a significant impact on improving human health status and longevity. In addition, there is scientific evidence, presented to the scientific community by Antoni et al. (2017) and Tinsley and Horne (2018), which has suggested that “fasting decreases body weight, insulin levels, blood pressure, inflammation, and appetite, and that it improves insulin sensitivity and lipid profiles,” which in many ways can also improve a human health status and longevity.

The previous paragraph described how fasting can improve a human health status and longevity in the general sense of the words. However, further analysis of this issue opens up new scientific questions that should be answered and offered to potential readers of this manuscript. Namely, it is important to emphasize that fasting is not in itself starvation but it represents a way of life that requires a certain sacrifice. For that reason, not all people are ready to approach it in the same way, so there are different types of fasting, each tailored to the individual in order to improve its health status and longevity. On the one hand, there are still religious priests, who fast in accordance with their centuries-old rules and maintain their health status at an adequate level, which leads to their longevity. On the other hand, there are a large number of people who are not able to organize their diet in accordance with the needs of their body. The modern lifestyle has led to the fact that their daily meals are not prepared in an adequate way, but “fast food” is taken. Meals are extensive and contain many non-nutritious ingredients, and are ingested both during the day and overnight, without any plan. Accordingly, the modern world has encountered a problem that is reflected in obesity. Namely, obesity is a serious problem, as it is associated with poorer mental health outcomes and reduced quality of life [NCD Risk Factor Collaboration (NCD-RisC), 2019]. Obesity is also linked to leading causes of death worldwide, including diabetes, heart disease, stroke and some cancers [NCD Risk Factor Collaboration (NCD-RisC), 2017]. For this reason, there are a large number of governmental and non-governmental organizations that show interest in the use of fasting for the treatment of obesity as it has been proven to be significantly correlated with the improvement of health status and longevity. However, there is no a general formula that can be suitable for each individual and different types of fasting have been defined over time. Next to the most recognized continuous energy restriction, it is interesting to present some alternative dietary weight loss strategies. Rynders et al. (2019) have created a strategy that “involves restricting energy intake to certain periods of the day or prolonging the fasting interval between meals (i.e., intermittent energy restriction)”. The strategy in question includes intermittent fasting and time-restricted feeding. Intermittent fasting is reflected to >60% energy restriction on 2–3 days per week, or on alternate days, and it covers “a broad class of interventions that alternate periods of eating and extended fasting, where it is associated with substantial weight loss in short periods of time, during around 8–12 weeks (Rynders et al., 2019).” According to the same authors, intermittent fasting interventions include “periodic 24-h fasts, intermittent energy restriction (e.g., the 5:2 diet), and time-restricted feeding that includes Ramadan fasting.” On the other hand, time-restricted feeding is “limiting the daily period of food intake to 8–10 h or less on most days of the week (Rynders et al., 2019).” Mattson et al. (2017) have created another strategy based on the duration. The strategy in question includes intermittent fasting and periodic fasting. According to mentioned authors, intermittent fasting suggests alternate day fasting (≥16 h) or 48 h of fasting/week, while periodic fasting suggests a minimum of 3 days of fasting every 2 or more weeks. Further analysis of the literature would lead to a number of other strategies, but the authors of this manuscript believe that the presented strategies are sufficient for each of the potential readers to choose one for themselves.

In summary, all the mentioned types of fasting in the previous paragraph have proven to be useful when it comes to improving human health status in many ways and, therefore, longevity. To make this claim more convincing, it is necessary, first of all, to refer to the research of Ravussin et al. (2019) who discovered that “time-restricted feeding has shown to facilitate weight loss through the meal-timing interventions by decreasing appetite rather than by increasing energy expenditure, where time-restricted feeding showed lowered values of ghrelin and leptin.” Also, the following three studies (Harvie et al., 2011; Gnanou et al., 2015; Panizza et al., 2019) confirm the assumptions that “intermittent energy restriction, combined by the specific diet have shown to generate a loss of body weight and fat mass,” while two more studies emphasized that “Ramadan fasting produced a weight loss through an increased energy expenditure (Lessan and Ali, 2019) and metabolic benefits (Fernando et al., 2019).” However, it is not enough to conclude this chapter only with recommendations related to limiting food intake, but it is important to note the part of the day in which food is consumed. Namely, it is a well-known fact that achieving an adequate muscle mass is a key factor for attaining a good body image, so Argiles et al. (2016) claim that “muscle is increasingly being recognized as a key tissue for the maintenance of an adequate health status.” The authors of this manuscript invite readers to pay special attention to proper nutrition during the period of food intake. They suggest there are a number of studies that recommend taking protein immediately after exercise, especially whey protein, which “acutely stimulates significant increases in post-exercise muscle protein synthesis (Atherton and Smith, 2012).” As proteins are widely used for active and sedentary people as a product for muscle growth, in addition to fasting, it is very important to use a diet rich in protein to lose weight and low in carbohydrates.

## Autophagy

Although the introductory part of this manuscript significantly deals with autophagy, it is still necessary to deal with another part of this topic, in order for potential readers to get a complete picture of this issue. Namely, it is very important to point out the conclusions of Thevelein et al. (2000) who emphasize that “aging cells gradually accumulate various deleterious changes, resulting in a decline in cellular function and eventually leading to cell death and disease.” Also, the same group of authors points out that “the accumulation of damaged macromolecules and organelles is one of the most persistent changes in aging cells,” while, Cuervo and Dice (1998) add that autophagy is “associated with the decline of different catabolic pathways.” Cuervo et al. (2005) in one of their recent studies concluded that the absence of autophagy in the elderly is one of the main causes that lead to the accumulation of biological “waste” reflected in damaged proteins and organelles. This group of authors based their assumptions on the facts obtained by Melendez et al. (2003), Kuma et al. (2004), as well as Juhasz et al. (2007) in their research on mice. These authors found that the loss of autophagy gene function led to the accumulation of damaged proteins and organelles within the cells, and that this accelerated the aging of mice, and thus shortened their lifespan. On the other hand, a study conducted by Simonsen et al. (2008) proved that elevating autophagy activity increases life span in the same species of mice, since the process of autophagy saved aged cells, so that there was no accumulation of dysfunctional mitochondria.

Autophagy is an evolutionarily conserved lysosomal catabolic process. In this process, the cells break down and recycle damaged organelles, incorrectly assembled proteins, then mutated proteins and macro molecules, as well as exogenous components such as bacteria and viruses. Therefore, autophagy is a process that aims to preserve cellular homeostasis in the human body. However, it is worth emphasizing that even autophagy cannot be viewed as a unique process characteristic of all types of activities that are initiated in that process. Namely, there are three main types of autophagy: (1) microautophagy, (2) chaperone-mediated autophagy and (3) microauthofagy. The specificity of each types of autophagy is reflected in the load type and the route of delivery to the lysosome. Mijaljica et al. (2011) defined microautophagy as a process that “involves the direct engulfment of cargo in endosomal/lysosomal membrane invaginations.” On the other hand, Kaushik and Cuervo (2012) described chaperone-mediated autophagy as a process that “recycles soluble proteins with an exposed amino acid motif that is recognized by the heat shock protein HSC70,” while macroautophagy, which is the best characterized type of autophagy, represents a process in which cytoplasmic constituents, which are encompassed by double-membrane vesicles called autophagosomes, fuse with lysosomes to form autolysis, and there the burden is degraded or recycled (Mizushima and Komatsu, 2011; Galluzzi et al., 2017).

In summary, autophagy is an essential process that takes place in human body with the goal of removing dysfunctional and unwanted parts of cells as well as regenerating existing cells to make them newer and healthier, so autophagy represents an evolutionary self-preservation mechanism through which humans can clean and repair its body (Marx, 2015). This explains the literal meaning of autophagy that is derived from the Ancient Greek for “self-eating”—“auto” means “self” and “phagy” means “eating.” From this reason, it should be easy to assume that autophagy should positively affect healthy longevity and well-being; however, there are some scientific studies to suggest that autophagy can have positive, but also negative health effects, so it is necessary to seek medical advice beforehand starting it (Schiattarella and Hill, 2016). Nevertheless, scientists still do not have a clear picture of all health implications of autophagy, so further researches are necessary, especially with the human subjects (most of researches have been conducted on non-human subjects). The part the scientists do know is connected to fact that the main benefits of autophagy are based on anti-aging principles and trying to turn the body clock back and create new and young cells. The scientists do also know that autophagy is increased when our cells are stressed, in order to protect human body and helps in the process of enhancing healthy longevity and well-being (Alirezaei et al., 2010). Although it is the fact that autophagy occurs naturally it is very important to set up a research question if the autophagy can be stimulated by some specific triggers. Some research strongly suggests that both fasting (intermittent fasting) and calorie restriction (ketogenic diets) can stimulate autophagy in the human body (Bagherniya et al., 2018), as well as, both regular physical activity (Brandt et al., 2018) and regular sleeping (Bedont et al., 2021).

## Physical Exercise, Sleep, Fasting and Autophagy: Practical Suggestions For Well-Being and Healthy Longevity

Movement is one of the basic needs of human being, which has sustained him during evolutionary development; however, most of the time the human being moved more than he needed to. In order to reduce the volume of heavy physical work, which was synonymous with the survival of human being for a long period of time, human being devised modern technologies that have taken over the role of human being in performing the aforementioned jobs. However, contemporary human being did not cope with the new circumstances, it can be said that human being even abused the ability to bring its movement and overall physical activity below the acceptable minimum necessary for the survival of human being as a healthy individual. If it is added to this the issue of the appearance of inorganic food on a large scale, it is clear that the diseases faced by contemporary human being and premature mortality are not a surprise.

Whether it will indulge in an inactive lifestyle and allow to be overwhelmed by the epidemic of immobility, but also all other epidemics that threaten us every day directly in front of or just around the corner, it is a main contemporary question. But it is a question that human population must not wait long to answer. There are solutions, but for them the common man must first find out and then persevere to get results, since the path to health and good energy requires certain sacrifices. Although autophagy is not an unknown term in scientific circles, it is increasingly becoming available to non-scientific audiences, especially after the guidelines that arrived from world-famous athletes, in the first place Novak Djokovic, the number one tennis racket. While waiting for stronger scientific confirmation, it is necessary to stick to the theories and knowledge that are currently available. So, the authors of this narrative review made recommendation, from the available practical and theoretical experience, to walk at least one kilometer every day, to sleep between seven and 9 h per night (for healthy adults), between seven and 8 h per night (for people over 65 years old), while in childhood and teens this period should be prolonged, not to take food for 16 h, just to drink exclusively water, tea and coffee without any additives (Pietrocola et al., 2014), between dinner at 6 pm and breakfast at 10 am the next day. On the other hand, the timetable does not need to be strict, so any individual can schedule any suitable timetable, just to keep 16 h food restrictions. For those who does not prefer to fast every day, the authors recommend a periodic fasting. This means minimum 3 days of fasting (the same food restrictions as described in the previous example) every 2 or more weeks.

Available theoretical knowledge and experience of individuals promise to raise every individual who consistently adheres to the above recommendations, in addition to biological benefits, and raising psycho-physical abilities, as well as the emergence of “youthful” energy, which gives a person the impetus to feel faster, stronger and more capable, much easier, enters into daily activities.

The fact is that a healthy, asleep and mobile citizen is a socially useful citizen. He represents a good producer, but also an equally useful consumer who, in the best way, maintains the economic chain at an adequate level. In such circumstances, that citizen is satisfied at home, satisfied on the street and satisfied at work, and society, with the majority of such citizens, is a welfare society, to which we should all aspire.

## Conclusion

This narrative review highlights the studies that explain regular physical exercise and sleep patterns, as well as fasting, and autophagy as a strategy for healthy longevity and well-being. Currently, any of these methods have been used for achieving healthy longevity and well-being within different stage of life from childhood to old-age; however, focusing on combination of all four methods instead of using just one should be the primary aim in the process of reaching healthy longevity and well-being in full capacity. Despite all the advances that have been made to create adequate physical exercise programs, sleep patterns or nutritional protocols, the relation between different types of fasting, nutritional supplementation and regular physical exercise and sleep patterns have not yet been satisfactorily resolved to cause the best effects of autophagy and, therefore, healthy longevity and well-being. Previous researches gave some guides how to create adequate protocols to reach the best effects of autophagy, but no studies answered the most important questions how to recognize the autophagy threshold and how to use various factors such as fasting and calorie restriction as well as regular physical activity and regular sleeping to stimulate autophagy and decrease the autophagy threshold. In this way, since there are no previous studies, the first future study should create a theory of autophagy threshold, while the rest of future studies should be clinical trials that would confirm independent and joint positive effects of regular physical exercises and sleep patterns, as well as fasting and autophagy on healthy longevity and well-being.

## Author Contributions

SM and BM formulated the research goals and aims, developed and designed the methodology, prepared the published work, and specifically wrote the initial draft. TB prepared the published work and specifically acquired the financial support for the project leading to this publication. RM, IV, MV, JL, JV, TF, BJ, RB, and SP prepared the published work, specifically with critical reviews, editing, and revisions. All authors commented on the draft and contributed to the final version, approved the publication of the manuscript, and agreed to be accountable for all aspects of the work.

## Funding

The study was supported by the Non-profit Central Research Institute Fund of Chinese Academy of Medical Science (Grant Number: 2020-JKCS-022).

## Conflict of Interest

The authors declare that the research was conducted in the absence of any commercial or financial relationships that could be construed as a potential conflict of interest.

## Publisher's Note

All claims expressed in this article are solely those of the authors and do not necessarily represent those of their affiliated organizations, or those of the publisher, the editors and the reviewers. Any product that may be evaluated in this article, or claim that may be made by its manufacturer, is not guaranteed or endorsed by the publisher.
